# Modes of Action of Herbal Medicines and Plant Secondary Metabolites

**DOI:** 10.3390/medicines2030251

**Published:** 2015-09-08

**Authors:** Michael Wink

**Affiliations:** Institute of Pharmacy and Molecular Biotechnology, Heidelberg University, INF 364, Heidelberg D-69120, Germany; E-Mail: wink@uni-heidelberg.de; Tel.: +49-6221-544-881; Fax: +49-6221-544-884

**Keywords:** plant secondary metabolite, bioactivity, evolutionary pharmacology, specific interactions, non-specific interaction, phytotherapy

## Abstract

Plants produce a wide diversity of secondary metabolites (SM) which serve them as defense compounds against herbivores, and other plants and microbes, but also as signal compounds. In general, SM exhibit a wide array of biological and pharmacological properties. Because of this, some plants or products isolated from them have been and are still used to treat infections, health disorders or diseases. This review provides evidence that many SM have a broad spectrum of bioactivities. They often interact with the main targets in cells, such as proteins, biomembranes or nucleic acids. Whereas some SM appear to have been optimized on a few molecular targets, such as alkaloids on receptors of neurotransmitters, others (such as phenolics and terpenoids) are less specific and attack a multitude of proteins by building hydrogen, hydrophobic and ionic bonds, thus modulating their 3D structures and in consequence their bioactivities. The main modes of action are described for the major groups of common plant secondary metabolites. The multitarget activities of many SM can explain the medical application of complex extracts from medicinal plants for more health disorders which involve several targets. Herbal medicine is not a placebo medicine but a rational medicine, and for several of them clinical trials have shown efficacy.

## 1. Introduction

Humans have always suffered from infections by bacteria, fungi, viruses and parasites, but also from inflammation, cold, digestive problems, pain and many other health disorders and diseases. Modern medicines, which are based on synthetic drugs and on antibiotics, have only become available during the last 150 years [1,2,3,4]. Previously, humans had to rely on drugs from nature, mostly from plants, but also from fungi and animals. Medicinal systems around the world, which had been developed thousands of years ago, heavily relied on herbal medicine; a good record of plants used is available for Traditional Chinese Medicine, Kampo medicine, Ayurvedic medicine, European medicine, and traditional medicines of Africa, Australia and Americas [5,6,7,8,9,10,11,12,13,14,15,16,17]. The treatment of infections and health disorders with herbal medicines is usually not or not entirely a placebo medicine but involves active natural products mostly of low molecular weight of great structural diversity (so-called secondary metabolites), which are typical for all plants [5,6,7,8,9,10,11,12,13,14,15,16,17,18,19,20].

### Why do Plants Produce so many Bioactive Metabolites?—A Lesson from Evolutionary Pharmacology

It is trivial but nevertheless an important observation that plants cannot run away or use active weapons when attacked by a plant eating animal (so-called herbivore), be it a mollusk, worm, insect or vertebrate. If challenged by microbes, vertebrates and human can rely on their highly effective innate and acquired immune system; such an immune system does not exist in plants. However, plants have been around for more than 400 million years on this planet and apparently survived albeit being challenged by herbivores and microbes. Thousands of structurally differing secondary metabolites have apparently evolved during plant development as a means for plants to defend themselves against herbivores and against bacteria, fungi and viruses [21,22,23,24,25,26,27,28,29,30]. Some SM also serve as signal compounds to attract pollinating and seed-dispersing animals, furthermore as antioxidants and UV protectants. From the point of view of evolutionary pharmacology, plant secondary metabolites represent an exciting library of bioactive compounds filtered by natural selection, which have been used by humans to treat infections and health disorders, or as spices, perfumes, arrow poisons, toxins and pesticides [25,31,32,33,34,35].

The challenge for pharmacology today is to describe and understand the diversity of SM, their modes of action alone or in natural combinations as found in plants. Another interesting task is to find out which plants have been used in traditional medicine systems around the world, to explore their phytochemistry and to explain if and how their SM may contribute to the recorded pharmacological activities. Especially in Europe, several of the traditional medicines have been developed into modern registered drugs which have been studied in clinical trials. For a number of these plant drugs controlled clinical trials have proven their efficacy and can thus be prescribed in evidence-based medicine [1,5,6,8,11,12,13,16,20,36,37,38]. For several hundred medicinal plants from around the world monographs have been published, in which the therapeutic evidence has been assembled in an organized fashion; important monographs are those of the German Commission E [39], the European Pharmacopeia (PhEur) [40], the European Scientific Cooperative on Phytotherapy (ESCOP) [41,42], the World Health Organization (WHO monographs), and of the European Medicines Agency—Herbal Medicinal Products (HMBC monographs) [12].

In this review, I have explored the diversity of plant secondary metabolites and explain their bioactivities and the underlying modes of action. In view of more than 100,000 known secondary metabolites [22,23,24,30,43,44,45,46,47,48] my analysis can only be exemplarily. Because for lack of space, most of the literature cited here are reviews or handbooks which contain the original citations. Due to the progress in molecular medicine, which is increasingly based on extensive genome and transcriptome analyses, more and more molecular targets (e.g., genes involved in health disorders) have been identified. Some of these new targets will become available for testing and drugs are needed which affect them. It is likely that SM (known and new ones) will represent an interesting library in this context. They can then either be used directly or as a lead for synthetic or semisynthetic derivatives in modern medicine.

## 2. General Modes of Action of Secondary Metabolites

A number of plants are well-known for their toxic or hallucinogenic properties [13,23,31,34,35,38,49,50,51]. Very often, these plants contain certain alkaloids, terpenoids or other SM which specifically modulate a corresponding molecular target in animals or humans. Such targets are often neuroreceptors, enzymes which degrade neurotransmitters, ion channels, ion pumps, or elements of the cytoskeleton (mostly tubulin or microtubules) [13,28,38,51,52,53,54]. Quite a number of these SM are presently been extracted from plants and are used in modern medicine as chemical entities with established applications (Table 1) [4,12,55,56,57]. These SM appear to be quite specific for a given target. We have speculated that their shape had been formed during evolution and selection by a process of “evolutionary molecular modeling” [51,52,54] in analogy to chemical molecular modeling in medicinal chemistry.

A closer analysis of plants used in phytotherapy reveals that most of them do not contain the compounds listed in Table 1. The phytomedicines employed in phytotherapy [2,6,7,8,13,14,15,16,17,18,19,39,40,41,42,52,58,59,60,61,62,63,64,65,66,67] are usually utilized as an extract (water or alcohol extracts, distillate, or essential oil), which contains dozens or even hundreds of SM often from several structural groups. In most cases, it was almost impossible to define a single SM, which could explain the bioactivity of the extract or its application in traditional medicine. It is likely that the activity of an extract can be due to synergistic interactions of several SM present, which cannot be detected when single compounds are evaluated alone [68,69,70,71,72,73,74,75,76,77,78,79,80,81]. Moreover, these extracts are often used to treat a broad spectrum of health disorders and not a single condition.

**Table 1:** Use and bioactivity of a few selective secondary metabolites which are applied as isolated compounds in medicine [12]; alkaloid (A), terpenoids (T) [11,12,13,14].

Plant Species	Substance (Class)	Mode of Action	Properties/Applications
*Aconitum napellus*	aconitine (A)	activates Na^+^ channels	analgesic
*Atropa belladonna*	l-hyoscyamine (A)	antagonist of mAChR	parasympathomimetic
*Camptotheca acuminate*	camptothecin (A)	inhibitor of DNA topoisomerase	tumour therapy
*Cannabis sativa*	tetrahydrocannabinol (T)	activates THC receptor	analgesic
*Catharanthus roseus*	dimeric Vinca alkaloids (A)	inhibit microtubule assembly	tumor therapy
*Chondrodendron tomentosum*	tubocurarine (A)	inhibits nAChR	muscle relaxant
*Cinchona pubescens*	quinidine (A)	inhibits Na^+^ channels	antiarrhythmic
*Coffea arabica*	caffeine (A)	inhibits phosphodiesterase and adenosine receptors	stimulant
*Colchicum autumnale*	colchicine (A)	inhibits microtubule assembly	gout treatment
*Cytisus scoparius*	sparteine (A)	inhibits Na^+^ channels	antiarrhythmic
*Digitalis lanata*	digitoxin, digoxin (T)	inhibits Na^+^,K^+^-ATPase	heart insufficiency
*Erythroxylum coca*	cocaine (A)	inhibits Na^+^ channels and reuptake of noradrenaline and dopamine	analgesic; stimulant
*Galanthus woronowii*	galanthamine (A)	inhibits AChE	Alzheimer treatment
*Lycopodium clavatum*	huperzine A (A)	inhibits AChE	Alzheimer treatment
*Papaver somniferum*	morphine (A)	agonist of endorphine receptors	analgesic, hallucinogen
*Physostigma venenosum*	physostigmine (A)	inhibits AChE	Alzheimer treatment
*Pilocarpus joborandi*	pilocarpine (A)	agonist of mAChR	glaucoma treatment
*Psychotria ipecacuanha*	emetine (A)	protein biosynthesis inhibitor	treatment of amebae infections; emetic
*Rauvolfia reserpina*	reserpine (A)	inhibits the uptake of noradrenalin into postsynaptic vesicles	hypertonia treatment
*Sanguinaria canadensis*	sanguinarine (A)	DNA intercalator	antibacterial, antiviral
*Strophantus gratus*	ouabain (T)	inhibits Na^+^, K^+^-ATPase	heart insufficiency
*Taxus brevifolia*	paclitaxel (taxol) (A)	inhibits microtubule disassembly	tumour therapy

How to explain the apparent broad-band activity of extract drugs? The plants used in phytotherapy are usually rich in phenolic compounds (flavonoids, phenylpropanoids, rosmarinic acid, catechins, tannins, polyketides), terpenoids (mono- and sesquiterpenes, iridoids, saponins) and polysaccharides (Table 2). They hardly contain toxic alkaloids, cyanogenic glucosides, grayanotoxins, cucurbitacins, cardiac glycosides or phorbol esters [10,11,12].

**Table 2:** Composition of extracts from medicinal plants used in traditional phytotherapy and their putative interactions [10,11,12].

Medicinal Plant/Drug	Phenolics *	Terpenoids *	Saponins *	Polysaccharides *	Covalent Interactions **
*Actaea (syn. Cimicifuga) racemosa*	++	++			
*Aesculus hippocastanum*	++		++		
*Allium sativum*	+				++
*Althaea officinalis*	+			++	
*Andrographis paniculata*	+	++			
*Arctostaphylos uva-ursi*	++				++
*Arnica montana*	++	++	+	+	+
*Boswellia sacra*		++	++	+	
*Calendula officinalis*	++	++	++	+	
*Centella asiatica*		+	++		
*Cistus creticus*	++	+			
*Crataegus monogyna*	++		+		
*Curcuma longa*	++	++		+	
*Cynara cardunculus*	++	++			+
*Echinacea purpurea*	++			++	
*Eleutherococcus senticosus*	++	++	++	+	
*Eucalyptus globulus*	+	++			
*Filipendula ulmaria*	++	+			
*Gentiana lutea*	++	++		+	
*Ginkgo biloba*	++	++			
*Glycyrrhiza glabra*	++		++		
*Harpagophytum procumbens*	++	++			++
*Hypericum perforatum*	++	++			
*Matricaria chamomilla*	++	++		+	+
*Mentha piperita*	+	++			
*Orthosiphon aristatus*	++	++	+		
*Panax ginseng*	+	+	++		
*Pelargonium sidoides*	++	++			
*Plantago lanceolata*	++	++	+	++	+
*Potentilla erecta*	++		++		
*Quercus robur*	++		+		
*Rhemannia glutinosa*		++		+	++
*Rosmarinus officinalis*	++	++	+		
*Salix alba*	++				
*Silybum marianum*	++				
*Urtica dioica*	++		+		
*Vaccinium macrocarpon*	++				
*Verbascum phlomoides*	++	+		++	+
*Vitex agnus-castus*	++	++			++
*Zingiber officinale*		++			

**Figure 1:**
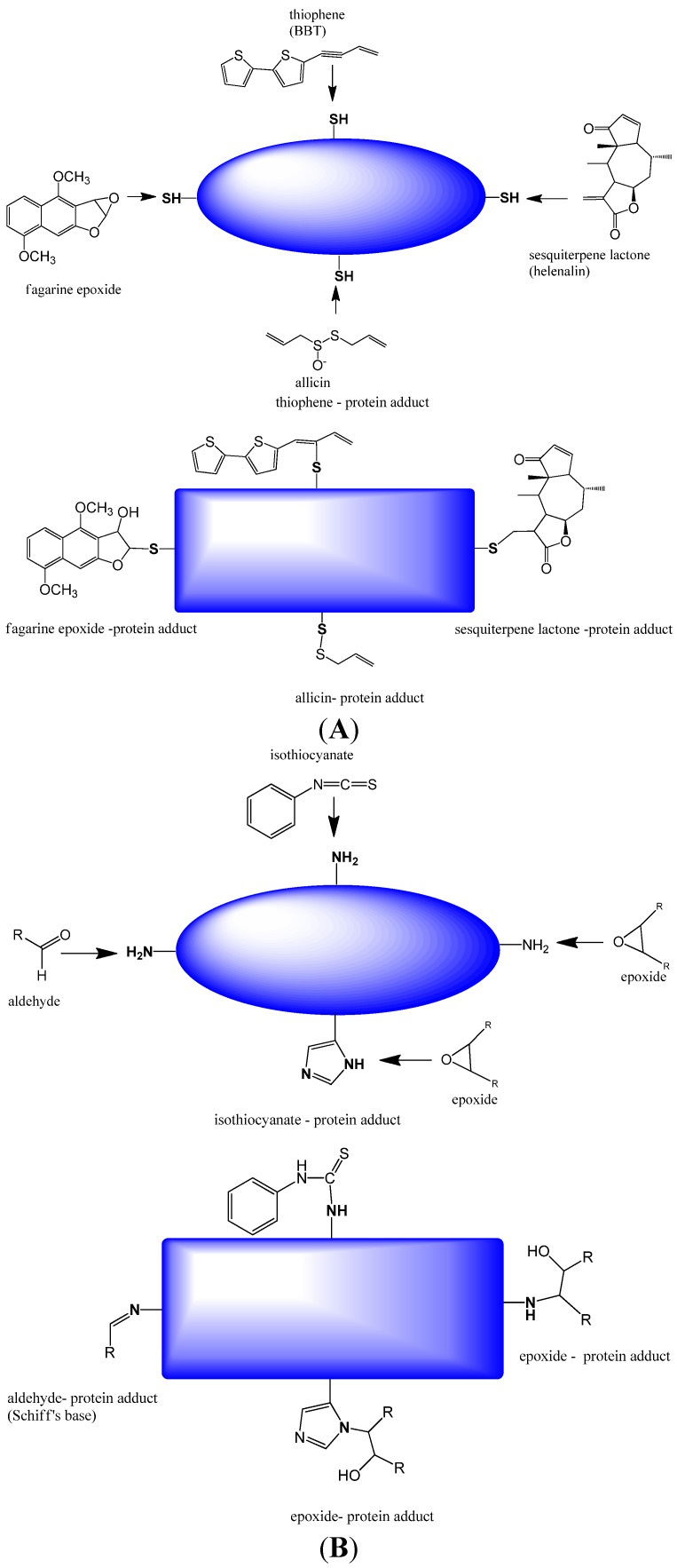
Secondary metabolites (SM) which form covalent bonds with proteins inducing a conformational change (indicated by the change of the form of the theoretical protein) and thus modulating their bioactivities (after [51,63]). (**A**) Interactions of SM with SH-groups of proteins and peptides; (**B**) Interactions of SM with amino groups of proteins or peptides.

**Figure 2:**
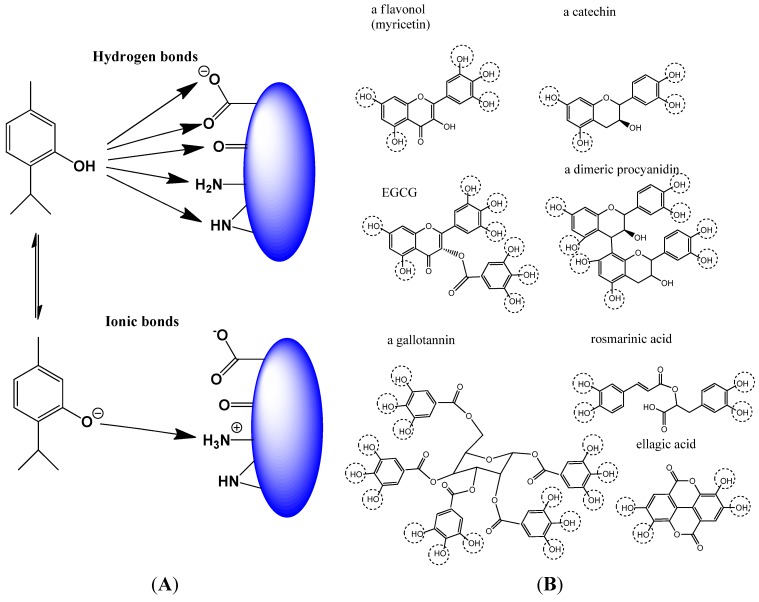
SM which form hydrogen and ionic bonds with proteins and thus modulate their conformation. (**A**) Schematic view of possible hydrogen and ionic bond formation by phenolic compounds (here thymol); (**B**) Examples for widely distributed polyphenols in medicinal plants. Phenolic OH groups are circled (after [51,63]).

**Figure 3:**
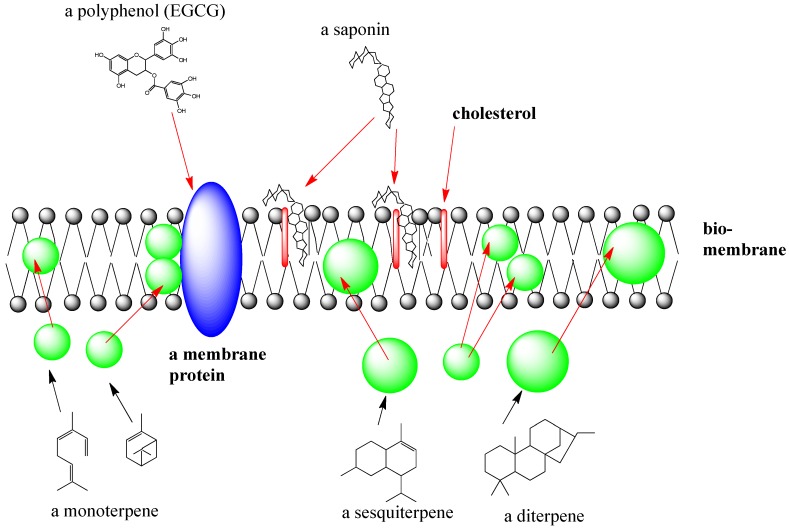
Interaction of SM with biomembranes. Saponins can complex membrane cholesterol; polyphenols influence 3D structure of membrane proteins (receptors, transporters, ion channels); small lipophilic terpenoids assemble in the inner lipophilic core of the biomembrane (after [11,12])

+: present; ++: main compounds; ** for covalent interactions see Figure 1; * for non-covalent interactions of phenolics, terpenoids and saponins see Figure 2 and Figure 3.

### 2.1. How Secondary Metabolites Used in Phytotherapy can Mediate Biological Activities?

#### 2.1.1. Covalent Modification of Proteins and DNA Bases

In general, plants not only produce the skeleton of a secondary metabolite but add a number of polar and non-polar substituents so that a library of SM of the same chemical class is present. Several SM contain very reactive functional groups in their structures (e.g., aldehyde and SH-groups, epoxides, double bonds with enon configuration, triple bonds) [11,12,13,23,25,38,44,45,51,59,60], which can form covalent bonds with proteins, peptides and sometimes DNA [61,62,63,64]. SM with an aldehyde group can establish a Schiff’s base with amino or imino groups of proteins, amino acid residues or DNA bases (Figure 1) under physiological conditions. Epoxides can easily react with free amino groups of proteins and DNA bases or SH-groups of proteins. Isothiocyanates (released from glucosinolates) can bind to amino and SH-groups. Exocyclic methylene groups (in terpenes, phenylpropanoids), allicin (from garlic) or sesquiterpene lactones can bind to SH-groups in proteins and glutathione. These modifications are formed almost at random under physiological conditions.

The most abundant target molecule of cells are proteins, which function as enzymes, receptors, transcription factors, ion channels, transporters, or cytoskeletal proteins. If receptors or enzymes are thus modified in their binding or catalytic site, they no longer can bind their ligand or substrate. However, the alkylation of proteins or peptides at other positions may also influence their 3D structures, which are important for protein-protein recognition, binding or catalytic activity or turnover. As a consequence SM with reactive functional groups can attack a multitude of proteins in an organism in a non-selective way; they can nevertheless be useful as “multitarget drugs” in diseases or health disorders in which many proteins are involved. Such drug would even target proteins whose activity has not been discovered as a relevant member of a signal pathway. If DNA bases become alkylated (e.g., by aldehydes or epoxides, but also by pyrrolizidine alkaloids, cycasin, aristolochic acid, furanocoumarins) mutations may be caused which might even lead to cancer. As a consequence, a number of mutagenic and carcinogenic compounds are known from nature [38,51,54]. Although sometimes used in traditional medicine, drugs with mutagens are usually obsolete in modern phytotherapy.

#### 2.1.2. Non-Covalent Modification of Proteins

Proteins, as the major target in cells are not only modulated by SM with reactive functional groups but also by phenols and polyphenols. Phenolic compounds, which are present in most herbal medicines (Table 2), carry one or several hydroxyl groups, which can form several hydrogen bonds with electronegative atoms (O, N) in peptides and proteins (Figure 2A,B) [11,12,13]. More importantly, phenolics carry one or several phenolic OH-groups, which can partly dissociate to negatively charged phenolate ions under physiological conditions [11,12,13,61,62,63] (Figure 2A).

These negatively charged groups readily form ionic bonds with positively charged amino groups of amino acid residues (e.g., in lysine, arginine) in proteins. If a SM, such as a polyphenol (Figure 2B) forms several hydrogen and ionic bonds with a protein or with its binding or catalytic site, the structural and functional flexibility of the protein becomes impaired. Similar to the situation of SM with reactive functional groups that form covalent bonds (Figure 1), also SM which make several hydrogen and ionic bonds, can affect proteins as multitarget drugs in a rather non-specific fashion. Phenolic compounds are often glycosylated with one or several sugar molecules. Since the carbohydrates carry several hydroxyl groups, they can further support the phenolics interaction with proteins by enforcing hydrogen bonding. Although the pleiotropic effects of polyphenols are well known, many papers have been published in which specific activities have been claimed for a particular target. If other protein targets would have been considered too, the more unspecific activity would have become more apparent.

An important class of proteins are transcription factors which regulate differential gene expression in an organism. Because also transcription factors can be modulated by phenolics or SM which form covalent bonds, gene regulation may be influenced indirectly. Indeed, whenever transcriptome analyses have been carried out in cells or animals treated with a herbal drug or even a single compound, many genes were found which were either up- or down-regulated [82,83,84]. With the availability of RNASeq using Next Generation Sequencing (NGS) it will become apparent that not only proteins are affected by SM but also several genes and also the proteins mediating differentiation and epigenetics.

#### 2.1.3. Interactions of SM with Biomembranes

All living organisms are surrounded by a semipermeable biomembrane which functions as a permeation barrier preventing the leakage of cellular metabolites into the surrounding but also the uncontrolled influx of external substances. Biomembranes also contain a multitude of proteins, such as ion channels, receptors and transporters which mediate a communication or the exchange of substances with other cells or tissues.

If a biomembrane is disturbed or lysed, usually necrotic cell death is a consequence. Many SM exist which have an affinity for biomembranes [11,12,13]. These are usually lipophilic SM. If they come into contact with a cell, the lipophilic compounds will bind to the lipophilic inner core of the membrane bilayer (Figure 3). This typically happens with several mono- and sesquiterpenes, which can assemble in membranes. If their concentration is high enough, this will change membrane fluidity and increase permeability. As a consequence, many lipophilic SM (especially those in essential oils) show antimicrobial and cytotoxic activities [11,12,13]. Lipophilic SM can also modulate the activity of ion channels; a well-known example is that of mint oil which affects calcium channels and the motility of smooth muscles cells in the intestines [11,12,13]. A special class of membrane active SM exist in saponins (see below) which can complex cholesterol in animal membranes and ergosterol in fungal membranes [11,12,13,23,24,85]. As a consequence of interactions of monodesmodisic saponins (with a single sugar chain) with cells, membranes are completely lysed. This activity can be easily demonstrated using red blood cells as they release the red hemoglobin into a buffer, when they are lysed by a saponin [11,12,13,23,77]. These membrane activities of certain SM are not specific but nevertheless quite powerful. Low concentrations of some saponins apparently enhance the uptake of polar SM, thus increasing their activity in an apparently synergistic fashion [23,69,70,75,76,77].

If polyphenols are present in an extract they can affect the activity of membrane proteins (Figure 3), which are already modulated by lipophilic SM which can disturb their interactions with phospholipids.

#### 2.1.4. Interactions of SM with Nucleic Acids

Some SM can intercalate or alkylate DNA, an activity which can cause mutations and even cancer. Important alkylating SM are pyrrolizidine alkaloids in Boraginaceae and some Asteraceae [86], aristolochic acids in *Aristolochia*, cycasine in cycads, furanocoumarins in Apiaceae, and ptaquiloside in *Pteridium aquilinum* [11,12,13,51]. Some alkaloids (e.g., sanguinarine, berberine) and furanocoumarins are both lipophilic, aromatic and planar which allows them to intercalate DNA [11,12,13,51,87]. DNA intercalation stabilizes DNA and can lead to frameshift mutations and after long-term use, to cancer. Some of the plants containing these potential carcinogens have been used in traditional medicine in many countries of the world because they show substantial antibacterial, antifungal, antiviral and cytotoxic properties [11,12,13,23,24]. In most countries regulations exists today which control marketing of such plants.

#### 2.1.5. SM with Antioxidant Properties

Reactive oxygen species (ROS) can react with important macromolecules of cells, such as proteins, lipids and nucleic acids. As a consequence an overdose of ROS may lead to several mostly chronic health disorders, such as diabetes, metabolic syndrome, cardiovascular disease and even cancer [13]. ROS may also influence the aging process.

Many phenolics, terpenoids with conjugated double bonds, and ascorbic acid are able to inhibit ROS and other oxygen radicals [11,12,13]. Many herbal drugs and products from algae rich in phenolics may therefore exhibit an antioxidant activity, in addition to modulation of proteins and biomembranes. An efficient *in vivo* model to study the effect of antioxidants against oxidative stress and aging is the model system *Caenorhabditis elegans*. For several herbal drugs and isolated polyphenols and terpenoids substantial antioxidant and antiaging effects have been recorded [88,89,90,91,92,93,94].

## 3. Which Secondary Metabolites Occur in Plants and how do They Function?

In Section 2 we discussed general modes of action of SM carrying functional groups or exhibiting certain general physicochemical properties. In the following, the main classes of common SM found in plants are introduced, and short information is provided for their occurrence, biological activity and pharmaceutical applications. From a biosynthetic perspective we can group SM into those without nitrogen or with nitrogen in their structures. Table 3 provides an overview of the main classes of SM and their biological functions.

**Table 3:** Estimated number of described secondary metabolites and their main functions for the plants producing them * (after [11,12])

Class	Numbers of Structures	Toxic or Repellent for Herbivores	Antimicrobial Activity	Attraction of Pollinators or Fruit Dispersers
**With nitrogen**				
Alkaloids	27,000	++++	++	−
Non-protein amino acids (NPAA)	700	++++	+++	−
Cyanogenic Glucosides/HCN	60	++++	+	−
Mustard oils (Glucosinolates)	150	++++	++++	+/−
Amines	100	+++	+	+++
Lectins, Peptides, AMPs	2000	+++	+++	−
**Without nitrogen**				
**Terpenes**				
Monoterpenes (including Iridoid glucosides)	3000	++	+++	+++
Sesquiterpenes	5000	+++	+++	++
Diterpenes	2500	+++	+++	−
Triterpenes, Steroids, Saponins (including cardiac glycosides)	5000	+++	+++	−
Tetraterpenes	500	+	+	+++
**Phenols**				
Phenylpropanoids, coumarins, lignans	2000	+++	+++	++
Flavonoids, anthocyanins, tannins	4000	+++	+++	++
Polyketides (Anthraquinones)	800	++++	+++	−
**Others**				
Polyacetylenes	1500	++++	++++	−
Carbohydrates, organic acids	600	+	++	+

* Activity: −: no SM active; +/−: very few SM active; +: few SM active; ++: many SM active; +++: most SM active; ++++: all SM active.

### 3.1. Nitrogen-Free Secondary Metabolites

#### 3.1.1. Terpenes

Terpenes are built from C5-units as a building block and can be subdivided into monoterpenes (C10), sesquiterpenes (C15), diterpenes (C20), triterpenes (C30), tetraterpenes (C40) and polyterpenes. Steroids (C27) are derived from triterpenes [23,43].

Most of the terpenoids are lipophilic. They readily interact with biomembranes and membrane proteins (Figure 3). As discussed in the last section, they can increase the fluidity and permeability of the membranes, which can lead to uncontrolled efflux of ions and metabolites and even to cell leakage, resulting in necrotic or apoptotic cell death [54]. In addition, they can modulate the activity of membrane proteins and receptors or ion channels. Some herbs with monoterpenes (e.g., *Mentha*) have therefore been used as a relaxant in case of spasm and cramps [11,12,13,24]. This membrane activity is rather non-specific. In general, terpenes show cytotoxic activities against a wide range of organisms, ranging from bacteria and fungi to insects and vertebrates and have been widely used in herbal medicine against infections [5,6,7,8,9,10,11,12,13,14,15,16,17,18,19,20,24]. Many terpenes are even effective against membrane-enclosed viruses. As shown in Table 2 terpenoids are widely present in extracts of medicinal plants.

##### Monoterpenes

Monoterpenes with an aromatic smell are widely present in Asteraceae, Apiaceae, Burseraceae, Dipterocarpaceae, Lamiaceae, Myricaceae, Myristicaceae, Poaceae, Rutaceae, Verbenaceae, and resin of conifers. In flowering plants they often serve to attract pollinating arthropods. They are isolated from plants in form of essential oils by distillation or solvent extraction [11,12,13].

Essential oils with monoterpenes are used in aroma therapy and in phytomedicine to treat rheumatism, infections (bacterial, fungal), cold, unrest, flatulence, intestinal spasms, as stomachic and to improve taste. Essential oils are ingredients of many perfumes and of some natural insect repellents. Applied to the skin, monoterpenes and aliphatic hydrocarbons can causes hyperemia; higher doses cause narcotic effects.

Thujone (in *Artemisia absinthium*, *Tanacetum vulgare*, *Thuja spec.*) contains a cyclopropane ring, which makes the molecule highly reactive [11,12,13,23]. Apparently, thujone can alkylate important proteins of the neuronal signal transduction, therefore causing neuronal disorder. This activity was the reason to ban the consumption of absinthe as a liqueur. Sabinen and sabinol are reactive monoterpenes in *Juniperus sabinus* with a highly reactive cyclopropane ring. Monoterpenes with exocyclic or terminal methylene groups, as in camphene, pinocarvone or in linalool, can bind to SH groups of proteins and thus change their conformation. Monoterpenes with a peroxide bridge, such as ascaridole, are reactive compounds, which can alkylate proteins [11,12,13,23].

Monoterpenes with phenolic hydroxyl groups (such as thymol and carvacrol) or with an aldehyde function (such as citral, citronellal) can bind to proteins (Figure 1 and Figure 2) and exhibit pronounced antiseptic properties; they are active against many bacteria and fungi.

##### Iridoid Glucosides

A subclass of monoterpenes are the iridoid glucosides with more than 200 structures distributed in the families Apocynaceae, Gentianaceae, Lamiaceae, Loganiaceae, Menyanthaceae, Plantaginaceae, Rubiaceae, Scrophulariaceae, Valerianaceae, and Verbenaceae [11,12,13,38]. Some of them, such as the gentiopicrosides, present in Gentianaceae and Menyanthaceae, exhibit an extremely bitter taste; they are used to improve digestion and to raise appetite in patients [11,12,13,24].

Iridoid glucosides, such as aucubin and harpagoside, are hydrolysed by a β-glucosidase into an unstable aglycone. Its lactol ring can open producing a functional dialdehyde [11,12,13,24]. Catalpol has a reactive epoxide ring in addition. The dialdehydes polygodial and warburganal have a peppery taste and have been recognized as the active principle in *Drymis aromatic*, *Polygonum hydropiper* and *Warburgia salutaris*. The dialdehyde can bind to proteins and form Schiff’s bases with free amino groups which appears to be the base for their pharmacological properties, which often included anti-inflammatory activities [11,12,13,24]. Several medicinal plants, rich in iridoid glucosides, have been used to treat infections, rheumatism and inflammations (*Harpagophytum procumbens*, *Plantago spec.*, *Scrophularia nodosa*, *Warburgia salutaris*). The secoiridoids in *Valeriana* contribute to the sedating properties of the medicinally used drug [11,12,13,24].

##### Sesquiterpenes and Sesquiterpenes Lactones

Sesquiterpene lactones (such as cyanarapicrin, helenalin, lactupicrin, parthenolide), which are common in Asteraceae and a few other families (Apiaceae, Magnoliaceae, Menispermaceae, Lauraceae, and ferns), can bind to SH groups of proteins via 1 or 2 exocyclic methylene groups (Figure 1) and the enon configuration in the furan ring and are therefore pharmacologically active, often as anti-inflammatory agents [11,12,13,24]. Some carry additional epoxide functions which make them more reactive. As discussed above, alkylated proteins can change their conformation and are no longer able to properly interact with substrates, ligands or other protein. Sesquiterpene lactones also bind glutathione (via SH groups) and can deplete its content in the liver and disturb the regulation of reactive oxygen species (ROS) in cells. As a consequence, sesquiterpenes lactones exhibit a broad range of biological activities, including cytotoxic, antibiotic, anthelminthic, anti-inflammatory, phytotoxic, insecticidal and antifungal properties [11,12,13,24].

Several plants with sesquiterpene lactones have been used in traditional medicine or phytotherapy (*Achillea*, *Arnica*, *Matricaria*, *Parthenium*) because they exhibit anti-inflammatory, expectorant, antibacterial, antifungal and antiparasitic properties [11,12,13,67]. The sesquiterpene artemisin with a reactive peroxide bridge from *Artemisia annua* has recently been developed into a potent antimalaria drug (artesunate), which is active against the dangerous *Plasmodium falciparum* [95].

##### Diterpenes

Several diterpenes are quite toxic, such as phorbol esters (present in Euphorbiaceae and Thymelaeaceae), which can be divided into those with a tigliane moiety and others with a daphnane or ingenane moiety [11,12,13,23]. The phorbol moiety carries one or two long chained esters so that the phorbol esters resemble diacyl glycerol a substrate of protein kinase C (PKC). Phorbol esters activate PKC and therefore cause severe inflammation; they are regarded as tumour promoters [11,12,13].

Plants with phorbol esters are strong purgatives; they induce drastic diarrhoea already after 5–10 min after ingestion. They are also potent skin irritants and lead to painful inflammation especially of mucosal tissue and of the eye. *Croton flavens* is an ingredient of Welensali tea which has been consumed in Curacao. A high incidence of oesophagus cancer has been recorded caused by welensalifactor F1. Some plants with phorbol esters (such as *Daphne mezereum*) have been used in traditional medicine as laxative and blister forming drug [11,12,13,38].

Another group of toxic diterpenes includes andromedotoxin (synonyms: acetylandromedol, grayanotoxin-I, rhodotoxin) and atractyloside. The neurotoxic andromedotoxin and related compounds are common in Ericaceae, especially in the genera *Gaultheria*, *Kalmia*, *Ledum*, *Pieris*, and *Rhododendron* [11,12,13,38]. The toxins can be transferred to honey by bees rendering it toxic. Andromedotoxins inhibit Na^+^ channels; they bind to receptor site II and block the transmission of action potentials. This causes bradycardia, hypotension and even death [11,12,13,38]. Atractyloside from *Atractylis gummifera* (Asteraceae) is a specific inhibitor of ADP transport across mitochondrial membranes and thus blocks energy supply of cells and organisms [38].

##### Triterpenes and Steroids

Some triterpenes and steroids show structural similarity with hormones (e.g., steroidal hormones, sex hormones, cortisone, ecdysone, juvenile hormone) and can thus modulate hormone responses in animals. A prominent example of bioactive steroids are the phytoecdysones, which have been isolated from ferns (*Polypodium vulgare*, *Pteridium aquilinium*) and several gymnosperms and angiosperms (*Achyranthes*, *Ajuga*, *Podocarpus*, *Rhaponticum*, *Silene*, *Vitex*) [38]. They mimic the insect moulting hormone ecdysone, stressing the role of terpenes for antiherbivore defense. In addition, mammalian steroidal sex hormones can be produced by plants, examples for estrogen producing plants are *Phaseolus vulgaris*, *Phoenix dactylifera*, *Punica granatum*, *Salix spec.* and for androgens the pollen of *Pinus sylvestris* [11,12,13,38].

Among steroidal glycosides, the cucurbitacins (occurring in members of the Cucurbitaceae and a few other families) express substantial cytotoxic activities; they inhibit tumor growth *in vitro* and *in vivo*. Cucurbitacins have been used to treat nasopharyngeal carcinoma. Cucurbitacins are highly cytotoxic as some of them may partially block mitosis in metaphase by inhibiting microtubule formation. Drugs with cucurbitacins have been used to treat malaria, as emetic or anesthetic (now obsolete), and in traditional medicine as diuretic, abortifacient and importantly as drastic laxative. Cucurbitacins irritate intestinal mucosa and cause release of water into the gut lumen. This in turn activates gut peristaltic and promotes diarrhoea. For topical use, *Bryonia* cucurbitacins have been applied to treat rheumatism and muscle pain [11,12,13,23,38].

##### Saponins

Saponins are the glycosides of triterpenes or steroids and include the group of cardiac glycosides and steroidal alkaloids. Steroid saponins are typical for several families of monocots, and are less frequent in dicots (Araliaceae, Fabaceae, Plantaginaceae, Scrophulariaceae, Solanaceae). Triterpene saponins are abundant in several dicot families, such as Ammaranthaceae (formerly Chenopodiaceae), Caryophyllaceae, Phytolaccaceae, Poaceae, Primulaceae, Ranunculaceae, and Sapotaceae. They are absent in gymnosperms [11,12,13,38].

In some cases, steroids, triterpenes and saponins structurally resemble endogenous anti-inflammatory hormones, e.g., glucocorticoids. The anti-inflammatory effects known from many medicinal plants could be due to a corticomimetic effect. A pronounced anti-inflammatory activity has been reported glycyrrhizic acid from *Glycyrrhiza glabra*, a triterpene saponin with sweet taste [11,12,13,38].

Some saponins are stored as bidesmosidic compounds (containing two sugar chains) in the vacuole, which are cleaved to the active monodesmosidic compounds by a β-glucosidase or an esterase upon wounding-induced decompartmentation. As described above (Figure 3), monodesmosidic saponins are amphiphilic compounds, which can complex cholesterol in biomembranes with their lipophilic terpenoid moiety and bind to surface glycoproteins and glycolipids with their sugar side chain [23,77,85]. This leads to a severe tension of the biomembrane and leakage. This membrane activity is rather unspecific and effects a wide set of organisms from microbes to animals. Therefore, saponins have been used in traditional medicine as anti-infecting agents [11,12,13,95,96]. Because saponins irritate the *Nervus vagus* in the stomach, which induces the secretion of water in the bronchia, saponin containing drugs are widely employed as secretolytic agents (*Hedera helix, Primula veris*) in phytomedicine [11,12,13,38].

Saponins have formerly been used as a detergent for washing clothes. Saponins are highly toxic for fish because they inhibit their respiration; therefore they have been traditionally employed for fishing. Saponins also kill water snails and have been employed to eliminate snails in tropical waters that transmit human parasites, such as *Schistosoma* (causing schistosomiasis) [23,38]. Steroidal saponins are important for the synthesis of steroid hormones (for the pill).

##### Cardiac Glycosides (CG)

Some saponins have additional functional groups, such as cardiac glycosides (carrying a 5 or 6 membered cardenolide or bufadienolide ring). Cardenolides have been found in Apocynaceae (*Apocynum*, *Nerium*, *Periploca*, *Strophanthus*, *Thevetia*, *Xysmalobium*), Brassicaceae (*Cheiranthus*, *Erysimum*), Celastraceae (*Euonymus*), Convallariaceae (*Convallaria*), Plantaginaceae (formerly Scrophulariaceae; *Digitalis*), and Ranunculaceae (*Adonis*). Bufadienolides occur in Asparagaceae (formerly Hyacinthaceae) (*Drimia*/*Urginea*), Crassulaceae (*Kalanchoe*), and Ranunculaceae (*Helleborus*) [11,12,13,23,38].

Although structurally different, all CG inhibit one of the most important molecular targets of animal cells, the Na^+^-, K^+^-ATPase, building up Na^+^ and K^+^ gradients which are essential for transport activities of cells and neuronal signaling. Therefore, cardiac glycosides are strong neurotoxins, which causes death through cardiac and respiratory arrest. Cardiac glycosides have been used in the past as arrow poisons. In medicine they are employed to treat patients with cardiac insufficiency. Cardiac glycosides slow down heart beat and exhibit positive inotropic, positive bathmotropic, weakly negative chronotropic and dromotropic heart activity. Isolated CG are still used to treat patients with cardiac insufficiency; in phytomedicine standardized extracts of CG producing plants are employed [3,11,12,13,23,24,38,54,55].

##### Tetraterpenes

Carotenoids represent the most important members of tetraterpenes. They are highly lipophilic compounds and are always associated with biomembranes. In chloroplasts they serve as accessory pigments important for photosynthesis. They also protect plants against UV light. Carotenoids in food and medicinal drugs are employed as powerful antioxidants [11,12,13,38]. Carotenoids are the precursors for vitamin A in animals, which is used to produce retinal (a light sensor in the rhodopsin complex) and retinoic acid (retinoids bind to nuclear receptors and are local mediators of vertebrate development). Some carotenoids are inhibitors of ABC transporters, which are often over-expressed in multidrug resistant cancer cells. If carotenoids are applied in combination with a cytotoxic drug, a reversal of drug resistance can be achieved [71].

##### Polyterpenes

Polyterpenes, consisting of 100 to 10,000 isoprene units, are prominent in latex of Apocynaceae, Asteraceae, Euphorbiaceae, Moraceae, and Sapotaceae. Some polyterpenes are used commercially such as rubber (from *Hevea brasiliensis*, Euphorbiaceae) or gutta-percha [11,12,13,38].

#### 3.1.2. Phenolics

Polyphenols (with several phenolic rings and phenolic OH groups) are present in most drugs used in phytotherapy (Table 2). As discussed in Section 2 phenolics apparently are responsible for a wide set of pharmacological properties, including antioxidant, anti-inflammatory, sedating, wound-healing, antimicrobial and antiviral activities [11,12,13,38].

##### Phenylpropanoids

Major lipophilic and aromatic phenylpropanoids include myristicin, safrol, eugenol, apiole, β-asarone, elemicin and estragole, which can be found in essential oils of Apiaceae, Myristicaceae, Rosaceae and several other families [11,12,13,38]. Phenylpropanoids with a terminal methylene group can react with SH groups of proteins (Figure 1). In the liver, these compounds are converted to epoxides, which can alkylate proteins and DNA (Figure 1). Therefore, they are potentially mutagenic and tumours have been observed in animal experiments. In particular, myristicin inhibits monoamine oxidase (MAO), which induces an increase of biogenic amine neurotransmitters, such as dopamine, serotonin and noradrenaline. Psychotropic effects resemble those of amphetamine. Eugenol is antiseptic and analgesic; it has been widely used in dentistry [11,12,13,23,24,38].

Medicinally important phenylpropanoids with a shortened side chain include salicylic acid, saligenin and the respective glucoside salicin. Because they inhibit a key enzyme of prostaglandin biosynthesis, *i.e.*, cyclooxygenase, they have been used in the treatment of inflammation, fever and chronic pain [3,11,12,13,23,24,38]. These compounds are known from willows (*Salix purpurea* or *S. alba*), *Filipendula ulmaria*, *Populus* spec*.*, *Primula veris*, and *Viola tricolor*.

Phenylpropanoids can also be conjugated with a second phenylpropanoid, such as in rosmarinic acid (Figure 2) or with amines, such as coumaroylputrescine. Rosmarinic acid (common in Lamiaceae) bears a number of phenolic hydroxyl groups with tannin-like activity (important for its anti-inflammatory, and antiviral activity) (Figure 2) [11,12,13,23,24,38]. Some phenols carry long alkyl and alkenyl side chains. Alkyl- and alkenyl phenols such as urushiol are abundant in Anacardiaceae, *Ginkgo*, Hydrophyllaceae, *Philodendron* and Proteaceae. Alkyl phenols are extremely allergenic compounds that are responsible for over a million poisoning instances (*Rhus* dermatitis) in USA. Contact with the eye is extremely hazardous; it can lead to blindness [11,12,13,38].

##### Coumarins and Furanocoumarins

Phenylpropanoids serve as building blocks for coumarins and furanocoumarins of which over 700 structures have been determined [43]. Coumarins can reach concentrations of up to 2% in plants and are common in certain genera of the Apiaceae (most genera), Fabaceae (e.g., *Dipteryx odorata*, *Melilotus officinalis*), Poaceae (e.g., *Anthoxanthum odoratum*), Rubiaceae (e.g., *Galium odoratum*). In phytomedicine they are used because of anti-inflammatory, anti-edemic and antimicrobial properties (*Melilotus*). Coumarins are aromatic and thus applied in cosmetics and in beverages [11,12,13,24,38].

Furanocoumarins (FC) usually have a third furane ring that is derived from active isoprene. Linear psoralen- or angular angelicin- type FC are distinguished. The furanocoumarins are present in aerial parts such as leaves and fruits but also in roots and rhizomes. They are abundant in Apiaceae (contents up to 4%), but also present in certain genera of the Fabaceae (e.g., *Psoralea bituminosa*) and Rutaceae [11,12,13,38]. The lipophilic and planar furanocoumarins can intercalate DNA and upon illumination with UV light can form cross-links with DNA bases, but also with proteins. They are therefore mutagenic and possibly carcinogenic. In medicine, furanocoumarins (such as 8-MOP) are employed for the treatment of psoriasis and vitiligo because FC can kill proliferating keratocytes in the skin upon UV exposure, this treatment brings some relieve for psoriasis patients [11,12,13,38].

##### Lignans and Lignin

Phenylpropanoids can form complex dimeric structures, so-called lignans. Podophyllotoxin, which occurs in members of the genera *Anthriscus* (Apiaceae), *Linum* (Linaceae), and *Podophyllum* (Berberidaceae), is a potent inhibitor of microtubule formation and thus prevents cell division. Pinoresinol and related compounds are inhibitors of cAMP phosphodiesterase, cytotoxic, insecticidal and immune modulating [11,12,13,23,38,51,54,97,98,99].

##### Flavonoids and Anthocyanins

Phenylpropanoids can condense with a polyketide moiety to flavonoids, stilbenes, chalcones, catechins and anthocyanins. These compounds are characterized by two aromatic rings that carry several phenolic hydroxyl or methoxyl groups [43,45]. In addition, they often occur as glycosides and are stored in vacuoles. Flavonoids are active ingredients of many herbal medicines [11,12,13,24,38].

The blue or reddish color of anthocyanins depends on the degree of glycosylation, hydrogen ion concentration and the presence of certain metals (e.g., aluminum ions) in the vacuole [43]. Anthocyanins are active antioxidants and are therefore used in phytomedicine or nutraceuticals to prevent ROS-related health disorders (*Aronia*, *Euterpe*, *Punica*, *Vaccinium*, *Vitis*) [23,91].

Stilbenes, such as resveratrol (present in red wine), have antioxidant, antibacterial and antifungal activities, and are present in several drugs and nutraceuticals [11,12,13,38]. Isoliquiritigenin and glyceollin II inhibit mitochondrial monoamine oxidase and uncouple mitochondrial oxidative phosphorylation. Rotenone inhibits mitochondrial respiratory chain and is therefore highly toxic and therefore used as an insecticide. The chalcone *O*-glycoside phloridzin from and *Kalmus*, *Pieris*, *Rhododendron* inhibits glucose transport at biomembranes [11,12,13,38]. The lignans from *Silybum marianum* (silybin, silandrin, silychristin) have antihepatotoxic properties and used to treat *Amanita* poisoning and liver cirrhosis [11,12,13,24,38]. Whereas many phenolics are bitter (e.g., naringin, neoeriocitrin, neohesperidin), some show sweet taste, such as the dihydroflavonols taxifolin 3-*O*-acetate, 6-methoxytaxifolin and 6-methoxyaromadendrin 3-*O*-acetate [11,12,13,38].

Isoflavones are common SM in legumes (subfamily Papilionoideae). They resemble the female sex hormone estradiol; they are therefore termed “phytoestrogens”. They can exhibit estrogenic and antioxidant properties and inhibit tyrosine kinases. Because of these properties they are often regarded as useful compounds that might play a role in the prevention of certain cancers, and for women with menopause or osteoporosis problems. Isoflavones from soy bean (*Glycine max*) and red clover (*Trifolium pratensis*) are marketed as nutraceuticals [11,12,13,38].

##### Catechins and Tannins

Catechins form a special class of flavonoids, which often dimerize or even polymerize to procyanidins and oligomeric procyanidins (Figure 2). The conjugates (which cannot be hydrolyzed; “non-hydrolysable tannins”) are characterized by a large number of hydroxyl groups. The phenolic hydroxyl groups can interact with proteins to form hydrogen and ionic bonds and possibly even covalent bonds (Figure 2). If more than 10 hydroxyl groups are present these compounds act as “tannins”. The tannin-protein interactions are a base for the utilization of plants with catechins in phytotherapy (e.g., *Crataegus monogyna* in patients with heart problems) [11,12,13,23,24,38].

Another important group of tannins is hydrolysable. They represent esters between gallic acid and sugars; in addition several moieties of gallic acid can be present that are also linked by ester bonds. These gallotannins are widely distributed in plants, often in bark, leaves and fruits. Gallotannins, which can additionally be condensed with catechins, contain a large number of phenolic hydroxyl groups so that they can form stable protein-tannin complexes and thus interact with a wide variety of protein targets in microbes and animals [11,12,13,38]. Tannins are strong antioxidants, with anti-inflammatory, antidiarrhoeal, cytotoxic, antiparasitic, antibacterial, antifungal and antiviral activities. Several medicinal plants (*Agrimonia*, *Alchemilla*, *Fragaria*, *Krameria*, *Potentilla*, *Quercus*, *Ribes*, *Sanguisorba*) are used internally and externally to treat inflammation and infection. They are common drug of traditional medicine and modern phytotherapy [11,12,13,23,24,38].

#### 3.1.3. Quinones

##### Quinones and Naphthoquinones

Quinones include hydro- and naphthoquinones and anthraquinones. Hydroquinones (such as arbutin) are typical for Ericaceae, naphthoquinones (such as droserone, juglone, plumbagin) for Balsaminaceae, Bignoniaceae, Droseraceae, Iridaceae, and Juglandaceae [11,12,13,38].

Quinones and naphthoquinones are redox reagents that can bind to enzymes or interact with proteins containing Fe^2+^/Fe^3+^, such as cytochromes and hemoglobin. Alkylated quinones can form novel antigens when bound to proteins and cause of dermatitis [23,38].

Drugs containing the antimicrobial arbutin are used in traditional medicine to treat bacterial infections of the urinary tract. A tea from *Tabebuia impetiginosa* (“Lapacho or Inka tea”), used by South American Indians, has been introduced in Europe as a general health tea and even for the treatment of cancer. Extracts from *Drosera* have been used in medicine as antitussive agents [11,12,13,23,24,38].

##### Anthraquinones and other Polyketides

Secondary metabolites with an anthracene skeleton can be present as anthrones, anthraquinones, anthranols, dianthrones, naphthodianthrones and dianthranoles [43]. Anthraquinones are characteristic for Asphodelaceae, Fabaceae, Hypericaceae, Liliaceae, Polygonaceae, Rhamnaceae, Rubiaceae, Scrophulariaceae, and Zanthorrhoeaceae [11,12,13,38]. Most anthraquinones carry phenolic OH groups and can therefore interfere with proteins similar to polyphenols (Figure 2), which can explain their broad activities.

Glycosylated monomeric anthrones target chloride channels, and Na^+^, K^+^-ATPase. In addition, the synthesis of a prostaglandin PGE2, histamine, and serotonin is stimulated and gastrointestinal hormones are released. Anthrones enhance peristalsis and the secretion of water and inhibit its absorption in the colon. Several anthraquinone containing drugs have been used for a long time (and are still employed) as a powerful purgative [11,12,13,23,24,38]. Since anthraquinones can intercalate DNA long-term usage is not encouraged [51]. Hypericin from *Hypericum* is stored in the skin by herbivores; upon exposition to UV light, a severe photodermatosis can occur. Special extracts from *Hypericum*, which contain hyperforin, flavonoids and/or hypericin serve as a powerful remedy against depression. Its efficacy has been demonstrated in several clinical trials [11,12,13,24,38].

#### 3.1.4. Polyacetylenes, Polyenes and Alkamides

Polyacetylenes or polyenes are aliphatic hydrocarbons with C-C triple and double bonds, such as in falcarinol [43,44]. Polyenes are common in Apiaceae, Araliaceae, Asteraceae, Campanulaceae, Oleaceae and Santalaceae [11,12,13,38]. Polyenes are reactive molecules that can interfere with membrane proteins (receptors, ion channels, transporters) and other proteins (Figure 1), especially by binding to SH groups. Most of them are active against bacteria, fungi, insects and nematodes. In *Tagetes* special polyenes are produced in which oxygen or sulfur have been added to the triple bonds and secondary ring formations have occurred (Figure 1). Typical examples are thiophenes from *Tagetes* that exhibit a wide range of antimicrobial and antiherbivore activities, some of which can be stimulated by light (phototoxicity).

Alkamides (150 structures have been reported) can be regarded as polyenes with nitrogen containing substituents. They occur in Aristolochiaceae, Asteraceae, Piperaceae, and Rutaceae; they appear to be antimicrobial, insecticidal and molluscicidal. They contribute to the immunostimulant activity of *Echinacea* [11,12,13,38].

#### 3.1.5. Carbohydrates

Plants produce and store several carbohydrates, most of which must be regarded as primary metabolites. Several carbohydrates, such as glucose, galactose or fructose are used to form glycosides with SM and are thus participants of both primary and secondary metabolism. Other carbohydrates appear to be allelochemicals in their own right: an example is phytic acid (a myo-inositol esterified with up to 6 phosphate groups) that can complex Ca^2+^ and Mg^2+^ ions and thus functions as an antinutritive substance [11,12,13,38].

Several di-, tri- and oligosaccharides, such as raffinose and stachyose (that are typical for seeds and roots) produce substantial flatulence and thus come closer to typical SM as they can be regarded as defense compounds against herbivores. Similar to the situation of *N*-containing defense chemicals in seeds, these oligosaccharides are additionally used as carbon source by the growing seedling.

Hexoses and pentoses are also building blocks for prominent polysaccharides of plants, *i.e.*, starch, cellulose, hemicellulose and pectin. In addition, a number of plants produce mucilage and specific storage products, such as inulin in Asteraceae and Campanulaceae which can be used medicinally for patients with diabetes. Plants rich in mucilage (*Althaea*, *Malva*, *Plantago*, *Verbascum*) are used in herbal medicine to treat cough, inflammation and to improve digestion [11,12,13,38]. Polysaccharides can interact with proteins and cell surfaced by forming several hydrogen bonds.

#### 3.1.6. Organic Acids

Most organic acids, such as acetic acid, fumaric acid, malic acid or citric acid have a prominent role in primary metabolism (Krebs cycle). In addition, fruits of many plants are rich in organic acids, which include those important in energy metabolism but also derivatives of them. In fruits they appear to carry ecological functions in preventing microbial infections or the feeding of immature fruits by herbivores. A number of organic acids derive from amino acids, such as senecic acid, angelic acid or tiglic acid, which are part of many SM in the form of esters [11,12,13,38].

Oxalic acid is a simple dicarboxylic acid, which can be present as a free acid or as a salt (e.g., water soluble potassium oxalate). In Araceae and Liliaceae oxalic acid is often deposited as hardly soluble calcium oxalate crystals that can form sharp needles (raphides), which makes such plants potentially toxic. Oxalic acid is a strong acid and powerful reducing agent. The sharp oxalate crystals of Araceae are potent irritants of skin and mucosal tissues; they can penetrate cells and cause necrosis [11,12,13,38]. The release of histamine causes itching, burning, salivation, and severe inflammation. Oxalic acid forms insoluble salts with calcium. If calcium oxalate is deposited in kidney tubules, kidney tissue becomes damaged. By depletion of calcium in the heart, the heart muscles can be damaged and its contractibility is reduced. In the blood, blood coagulation is also hampered by Ca^2+^ depletion. Plants with oxalic acid, which is a strong antioxidant, have a sour taste and some are consumed as vegetables, such as rhubarb or sorrel [11,12,13,38].

##### Ranunculin and Tuliposide

Ranunculin is a characteristic SM of Ranunculaceae, which is converted to the reactive protoanemonine after enzymatic cleavage. Tuliposide (releases tulipalin) has been found in the genera *Alstroemeria*, *Bornarea*, *Erythronium*, *Fritillaria*, *Gagea*, *Notholirion*, *Lilium*, and *Tulipa*. Tulipalin and protoanemonine have a highly reactive extracyclic methylene group that can form covalent bonds with free sulfhydryl groups of proteins or glutathione (Figure 1) [11,12,13,38]. Therefore, cytotoxic and allergenic effects can occur. Protoanemonine can also alkylate DNA and is therefore mutagenic. It exhibits antibacterial and antifungal properties. Tuliposide and tulipalin have cytotoxic and fungitoxic properties. Albeit their toxicity, some plants with protoanemonine are used in traditional medicine (*Pulsatilla*, *Anemona*) to treat infections and cold [11,12,13,38].

### 3.2. Nitrogen-Containing Secondary Metabolites

#### 3.2.1. Alkaloids (Including Amines)

Alkaloids are among the most active secondary metabolites and widely distributed in the plant kingdom (especially in angiosperms). Their structures contain one or several nitrogen atoms either in a ring structure (true alkaloids) or in a side chain (pseudoalkaloids). Depending on the ring structures, alkaloids are subdivided into several subgroups [43,100].

Alkaloid are infamous as animal toxins and certainly serve mainly as defense chemicals against predators (herbivores, carnivores) and to a lesser degree against bacteria, fungi and viruses. As discussed above, the molecular targets of alkaloids and amines often are neuroreceptors, or they modulate other steps in neuronal signal transduction, including ion channels or enzymes, which take up or metabolize neurotransmitters or second messengers [11,12,13,23,27,38,52,101,102,103,104]. Other alkaloids are mutagenic in that they intercalate or alkylate DNA [38,53,54,87]. Several alkaloids which interfere with DNA, telomeres, telomerase, topoisomerase, the cytoskeleton or protein biosynthesis induce apoptosis [51,53,57,62,70,82,105,106,107,108,109,110,111,112]. Some of them are used in cancer therapy as chemotherapeutics, such as Vinca alkaloids, paclitaxel or camptothecin (Table 1) [4,13,14,23,105,113,114,115].

A number of lipophilic alkaloids and other SM are substrates of ABC transporters, such as p-gp which are often overexpressed in cancer cells, parasites and microbes [81,114]. A strategy to overcome multiresistant cancer or microbial cells could be the combination of a chemotherapeutic drug with an inhibitor of ABC transporters. *In vitro* this strategy is powerful [54,62,67,69,70,71,77,78,80,81,82,99,116,117,118,119] but less *in vivo*.

##### Amaryllidaceae Alkaloids

Typical alkaloids in this group are ambelline, galanthamine, haemanthamine lycorine, and narciclasine, which are produced by several genera of the Amaryllidaceae. Lycorine and narciclasine inhibit ribosomal protein biosynthesis by binding to the 60S subunit [11,12,13,23,24,38]. Galanthamine, which is isolated from *Galanthus woronowii*, *Leucojum aestivum*, *Narcissus pseudonarcissus* and *N. nivalis*, has been introduced as a therapeutic to treat Alzheimer’s disease because it inhibits cholinesterase (as a parasympathomimetic) (Table 1). In addition, it shows analgesic properties [11,12,13,23,24,38].

##### Bufotenin, Tryptamines and Tyramines

Bufotenin occurs in the legumes *Anadenanthera peregrina*, *Banisteriopsis rusbyana* (Malpighiaceae), and *Mucuna pruriens*, but also in skins of toads. *N,N*-Dimethyltryptamine is produced by some mimosoid legumes, *Banisteriopsis argentea* (Malpighiaceae) and *Virola peruviana* (Myristicaceae). Hordenine and other tyramines have been found in Cactaceae and in Poaceae (*Hordeum*, *Phalaris*). Psilocin and its phosphate ester psilocybin are common ingredients of sacred and hallucinogenic mushroom of Mexico “Teonanacatl” (*Psilocybe mexicana*; Strophariaceae) [11,12,13,23,24,31,34,38].

The methylated tryptamines are analogues of the neurotransmitter serotonin (5-hydroxytryptamine) and thus work as 5-HT agonists. They stimulate 5-HT receptors, which evokes psychedelic hallucinations and euphoric feelings. Extracts from plants and mushroom with these psychoactive amines have been used as mind-altering drugs [11,12,13,23,24,31,34,38].

##### Colchicine

Colchicine and related alkaloids are typical SM of plants in the genera *Colchicum*, *Gloriosa* and a few other Liliaceae. The molecular target of colchicine is tubulin; it inhibits the polymerization of tubulin and depolymerization of microtubules which are necessary for cell division and intracellular transport of vesicles [38,54]. Colchicine inhibits the synthesis of collagen and activates collagenase. Colchicine has been used against fast dividing cancer cells, but its toxicity prevents a general application. In modern medicine colchicine is prescribed in cases of acute gout as it prevents macrophages from migrating to inflamed joints [3,11,12,13,23,24,38].

##### Diterpene Alkaloids

Aconitine from *Aconitum spec.* and protoveratrine B from *Veratrum spec.* are potent activators of Na^+^ channels that are essential for neuronal signaling. If these ion channels are completely activated, the action potential from nerves to muscles are no longer transmitted leading to a complete arrest of cardiac and skeletal muscles. Aconitine and protoveratrine B first activate and then paralyze the sensible nerve endings and neuromuscular plates. Aconitine also exerts analgesic properties and has been used to treat neuronal pain, such as caused from irritation of the trigeminus nerve. Extracts from *Aconitum* have been widely used as arrow poison, deadly poison and in witch ointments for thousands of years in Europe and Asia [11,12,13,23,24,38,52].

Another diterpene is paclitaxel (taxol^®^) that can be isolated from several yew species (including the North American *Taxus brevifolia*). In the Eurasian *T. baccata* taxanes are produced in the leaves which can be converted into paclitaxel. Paclitaxel stabilizes microtubules and thus blocks cell division in the late G2 phase; because of these properties, paclitaxel has been used for almost 20 years with great success in the chemotherapy of various tumors [3,11,12,13,14,23,24,38,51,54,55,113,114,115].

##### Ergot Alkaloids (EA)

Two series of EA can be distinguished: the clavine alkaloids (agroclavine and elymoclavine) and lysergic acid amides (ergine, ergometrine and more complex peptide alkaloids, such as ergotamine and ergocristine) [11,12,13,23,24,38]. EA are produced by a symbiotic fungus *Claviceps purpurea*, and more than 40 further members of this genus which exist as symbionts on grasses (tribes Avenae, Agrosteae, Festucaceae, Hordeae). Rye is especially affected among cereals. Ergot alkaloids are also common SM of some genera of the Convolvulaceae (including *Argyreia*, *Ipomoea*, *Rivea corymbosa*, *Stictocardia tiliafolia*) which carry the fungi as endophytes [11,12,13,23,24,26,38].

EA modulate the activity of noradrenaline, serotonin and dopamine receptors as agonists, partial agonists but also antagonists [52]. Consequences are contraction of smooth muscles of peripheral blood vessels (causing gangrene), or permanent contraction of uterine muscles, causing abortion. By blocking α-adrenergic receptors the alkaloids can induce the relaxation of smooth muscles (spasmolysis). The alkaloids inhibit serotonin receptors but stimulate dopamine receptors. Ergometrine (an α-receptor agonist) and derivatives are used in obstetrics to stop bleeding after birth or abortion and ergotamine (antagonist at noradrenaline and 5-HT receptor; agonist at dopamine receptor) to treat migraine. Ergocornine reduces the secretion of prolactin and inhibits nidation as well as lactation. LSD (*N,N*-diallyllysergic acid amide), which is a synthetic derivate of ergot alkaloids, is one of the strongest hallucinogens [11,12,13,23,24,38].

Poisoning with EA contaminated meal flour cause the dramatic and cruel effects of ergotism which has been documented in many paintings of the Old Masters. The hallucinogenic Mexican drug “ololiuqui” is composed of EA from *Ipomoea argyrophylla*, *I. violacea*, other *Ipomoea* species and *Rivea corymbosa* [11,12,13,23,24,38].

##### Indole Alkaloids (including Monoterpene Indole Alkaloids)

Indole alkaloids occur mainly in four plant families—the Apocynaceae, Gelsemiaceae, Loganiaceae, and Rubiaceae. Many of them show strong biological activities [11,12,13,23,24,38]. Ajmaline from *Rauvolfia serpentina* blocks sodium channels and has therefore antiarrhythmic properties because it lowers cardiac excitability. It has negative inotropic properties and is used medicinally to treat tachycardial arrhythmia, extra systoles, fibrillation and angina pectoris. Ajmalicine from *Rauvolfia serpentina* has a pronounced dilatatoric activity in blood vessels, which causes hypotension. Ajmalicine is used as a tranquillizer, antihypertensive to improve cerebral blood circulation. Toxiferine I and II from *Strychnos* are neuromuscular blocking agents, thus highly toxic and used as an arrow poison. They are strong inhibitors of nicotinic AChR at the neuromuscular plate and cause paralysis of muscle cells. Ibogaine from *Tabernanthe iboga* is a CNS stimulant with anticonvulsant and hallucinogenic properties. Physostigmine, eseridine and related compounds from *Physostigma venenosum* are strong inhibitors of cholinesterase with wide ranging parasympathetic activities. Physostigmine is used as a miotic in eye treatments and in the therapy of Alzheimer. Physostigmine is highly toxic and calabar beans were used as an ordeal poison in West Africa [11,12,13,23,24,38].

Harman or β-carboline alkaloids occur among others in Malpighiaceae (*Banisteriopsis*), Rutaceae (*Clausena*, *Murraya*) and Zygophyllaceae (*Peganum*, *Zygophyllum*). β-carboline alkaloids are inhibitors of MAO and agonists at serotonin receptors. Since they enhance serotonin activity, they exhibit substantial hallucinogenic activities and might be useful to treat patients with depression [11,12,13,23,24,38]. Harmine can interfere with telomeres and telomerase in cancer cells [112].

Dimeric Vinca alkaloids (vincristine, vinblastine, leurosine) from *Catharanthus roseus* inhibit tubulin polymerization and intercalate DNA (Table 1). As a consequence they effectively block cell division and are therefore important drugs used in cancer therapy [11,12,13,23,24,38,54,113,114]. Camptothecin, an inhibitor of DNA topoisomerase used in cancer therapy, is mainly produced from *Camptotheca acuminata* (but is also found in some genera of Apocynaceae, Gelsemiaceae, Icacinaceae, Rubiaceae) [11,12,13,23,24,38,54,113,114].

Reserpine and related alkaloids from *Rauvolfia serpentina* inhibit transporters for neurotransmitters at vesicle membranes and thus acts as an antihypertensive and tranquillizer. Strychnine from *Strychnos nux-vomica* is an antagonist at glycine gated chloride channel. It is a CNS stimulant and extremely toxic. Mesembrine, a simple indole alkaloid from *Sceletium*, is a narcotic with cocaine like activities has been used as an antidepressant. Gelsemine and gelsemicine from *Gelsemium* are CNS active and highly toxic [11,12,13,23,24,38,52].

##### Isoquinoline Alkaloids (including Protoberberine, Aporphine, and Morphinane Alkaloids)

Isoquinoline alkaloids are common in genera of the Annonaceae, Berberidaceae, Magnoliaceae Monimiaceae, Menispermaceae, Lauraceae, Papaveraceae, Ranunculaceae, Rutaceae and others. Many protoberberine and benzophenanthridine alkaloids interfere with neuroreceptors and DNA (several are strong DNA intercalators). The intercalating alkaloids (such as berberine, sanguinarine) show pronounced antibacterial, antiviral and cytotoxic properties [11,12,13,23,24,38,51,54,81].

*Chelidonium majus* has been used in traditional medicine and phytomedicine as cholagogue, spasmolytic, diuretic and analgesic drug or to treat warts. Chelidonine has been employed as a painkiller to treat abdominal pain, and to treat spasms and asthma. Extracts of *Sanguinaria canadensis* which are rich in the DNA-intercalating benzophenanthridine alkaloid sanguinarine have been included in mouth washes and toothpaste [11,12,13,23,24,38,82]*. Chelidonium* extracts and isolated alkaloids (berberine, sanguinarine) are cytotoxic in several cancer cell system and they inhibit ABC transporters [83].

Extracts of *Eschscholzia californica*, which are rich in aporphine, benzophenanthridine and protoberberine alkaloids, have been employed as a mild psychoactive drug to induce euphoria. The aporphine boldine (from *Peumus boldo*) is used to treat hepatic dysfunction and cholelithiasis. Emetine and cephaeline from (*Psychotria ipecacuanha*) have been used as emetic, expectorant and anti-amoebic. Cepheranthine, a bisbenzylisoquinoline from *Stephania*, has been used to treat tuberculosis and leprosy. Erythrina alkaloids block signal transduction at the neuromuscular plate and have been used as curare substitute [11,12,13,23,24,38]. Tubocurarine and other bisbenzylisoquinoline alkaloids from *Chondodendron* and *Ocotea* have been used traditionally as arrow poison but also in surgery as muscle relaxant (inhibition of nAChR). Papaverine (from several *Papaver* species) inhibits phosphodiesterase and thus acts as smooth muscle relaxant, vasodilator, and spasmolytic [11,12,13,23,24,38,52].

Morphinane alkaloids are typical for members of *Papaver somniferum*, and *P. bracteatum:* Morphine causes central analgesia, euphoria, and sedation. Morphine is an agonist of endorphine receptors in the brain and other organs and promotes powerful sleep-inducing, analgesic and hallucinogenic effects [11,12,13,23,24,38,52]. It is used in standardized modern medicines intended for oral and parenteral use—mainly to treat intense pain (e.g., in cancer patients) [3,54]. Codeine is an effective painkiller (though less active than morphine, but also less addictive); it sedates the cough center and is widely used as antitussive agent. Morphine and other morphinane alkaloids show addictive properties [11,12,13,23,24,38].

##### Phenylpropylamines

This group of bioactive amines with pronounced pharmacological activity, includes cathinone (from *Catha edulis)*, ephedrine (from several *Ephedra* species) and mescaline (*Lophophora williamsii* and other Cacti). Cathinone and ephedrine structurally resemble amphetamines and act in a similar way as sympathomimetics. These alkaloids stimulate α- and β-adrenergic dopaminergic receptors by stimulating the release of noradrenaline and dopamine from catecholic synapses and inhibiting their re-uptake [11,12,13,23,24,38,52]. Ephedrine causes vasoconstriction, hypertension, bronchial dilatation, and heart stimulation. Plants with ephedrine or cathinone reduce hunger sensation and have been used as appetite depressant and stimulant. Ephedrine has been used medicinally to treat asthma, sinusitis and rhinitis. Mescaline is a psychomimetic; it is a CNS depressant and hallucinogenic in high doses [11,12,13,23,24,38].

##### Piperidine Alkaloids

Piperine is the pungent principle of *Piper nigrum* and other *Piper* species. *Piper* fruits are widely used as hot spice and sometimes as insecticide. Piperine inhibits ABC transporter [117]. Coniine is a famous toxin from *Conium maculatum*. It causes ascending paralysis, which starts at the tip of arms and legs and ends with respiratory failure and death. Conium alkaloids are extremely toxic and teratogenic in livestock. Arecoline and arecaidine from *Areca catechu* exhibit parasympathetic activities and acts as a central stimulant widely used in SE Asia [11,12,13,23,24,38]. Lobeline occurs in *Lobelia* spec*.* and has been used in the treatment of asthma and as anti-smoking drug; it inhibits ABC transporter [119]. Pelletierine from *Punica granatum* has been used against intestinal tapeworms [11,12,13,23,24,38].

##### Purine Alkaloids

Caffeine, theophylline and theobromine are produced by *Camellia sinensis*, *Coffea arabica*; *Cola acuminata*, *Cola nitida,* *Ilex paraguarensis*, *Paullinia cupana*, and *Theobroma cacao.* Purine alkaloids function as CNS stimulants conferring wakefulness and enhanced mental activity. Caffeine inhibits cAMP phosphodiesterase and adenosine receptors [11,12,13,23,24,38,52]. As a consequence dopamine is released and many brain parts become activated. These alkaloids are a cardiac stimulants, vasodilators and smooth muscle relaxants. Extracts with purine alkaloids are widely used by humans as stimulants; caffeine is incorporated into numerous formulations employed against fever, pain, and flu symptoms [11,12,13,23,24,38].

##### Pyrrolidine Alkaloids

Nicotine (from *Nicotiana tabacum*) is an agonist at nACh-receptors and functions as a CNS stimulant with addictive and tranquillizing properties [11,12,13,23,24,38,52]. Today also used in electric cigarettes (“E-cigarette”). Before the availability of synthetic insecticides, nicotine was widely used as a natural insecticide in agriculture [13].

##### Pyrrolizidine Alkaloids (PA)

PA (such as senecionine, heliotrine) are produced from nearly all members of the Boraginaceae [86], several Asteraceae (subfamily Senecioninae) and Fabaceae (tribe Crotalarieae). PA are activated in the liver of humans or animals to reactive pyrroles (dehydropyrrolizidines) that can alkylate DNA-bases. These DNA alkylations can lead to mutation and cell death (especially in the liver). Furthermore, mutations can lead to malformations in pregnant animals and humans, and to cancer of liver, kidneys and lungs [11,12,13,23,24,38]. PA also modulate several neuroreceptors [21], which can explain their short-term repellence against herbivores.

Several PA-containing plants are used in traditional phytomedicine to treat bleeding or diabetes or general as herbal-tea (*Crotalaria*, *Heliotropium*, *Petasites*, *Senecio*); *Symphytum officinal*e and other Boraginaceae to treat wounds, broken or injured bones. Others, such as comphrey *Symphytum x uplandicum* are regularly supplied on local markets as “healthy” salad ingredients. Drugs containing PA are banned as medicines [11,12,13,23,24,38].

##### Quinolizidine Alkaloids (QA)

QA (such as anagyrine, cytisine, lupanine, sparteine) are common secondary metabolites in genistoid legumes (Fabaceae). They affect acetylcholine receptors and ion channels; they are poisonous neurotoxins for animals. Sparteine from *Cytisus scoparius* has been employed medicinally to treat heart arrhythmia (Na^+^ channel blocker) and during child birth (inducing uterus contraction). Plants with anagyrine can cause malformations (“crooked calf disease”) if pregnant animals feed on plants (such as lupins) containing it [11,12,13,23,24,38,52,102].

##### Quinoline Alkaloids (including Acridone Alkaloids)

Medically important quinolone alkaloids (occurring in Acanthaceae, Rubiaceae, Rutaceae) include quinine, quinidine and cinchonidine, which have been used as antimalarial [67]. Quinidine which inhibits Na^+^ channels has antiarrhythmic properties. Quinine is very bitter and is employed as a bittering agent in food industry. Peganine and vasicine (and related compounds) show cholinergic activity. Most quinoline alkaloids intercalate DNA and thus cause frame shift mutations [11,12,13,23,24,38,51]. Furanoquinolines can be activated by light and can form covalent bonds with DNA bases. This explains their cytotoxicity, antibacterial and antifungal properties. When human skin that has been in contact with furanoquinolines, such as fargarine, dictamnine or skimmianine, and is exposed to sun light severe burns can occur with blister formation, inflammation and necrosis [11,12,13,23,24,38,51].

##### Steroid Alkaloids

Steroidal alkaloids which often consist of a lipophilic steroid moiety and a hydrophilic oligosaccharide chain (as in saponins) are produced by four unrelated plant families: Apocynaceae, Buxaceae, Liliaceae and Solanaceae. They are especially widely distributed within the very large genus *Solanum* that includes potato, tomato and other food plants produce the spirosolane type, with soladulcidine and tomatidine and solanidane type with solanine and chaconine [11,12,13,23,24,38,43].

Solanum alkaloids behave as saponins (see under saponins). This property also explains the strong skin irritation seen on mucosa and the antifungal properties and cytotoxic activities known from saponins. In addition, the alkaloids inhibit acetylcholine esterase that breaks down acetylcholine in the synapse. Therefore the *Solanum* alkaloids cause some neuronal effects [11,12,13,23,24,38,52]. Several *Solanum* species, such as *Solanum dulcamara* are part of traditional medicine used as anti-inflammatory drugs. Solanum alkaloids have been used in agriculture as an insecticide. Plants of the genus *Buxus* contain a series of free steroidal alkaloids, such as cyclobuxine D, buxamine E, which are quite toxic and strongly purgative [11,12,13,23,24,38].

##### Tropane Alkaloids (TA)

TA, such as l-hyoscyamine (or its racemate atropine) and l-scopolamine are common SM in several genera of the Solanaceae (*Atropa*, *Datura*, *Duboisia*, *Hyoscyamus*, *Mandragora*, *Physalis*, *Physoclaina*, *Salpichroa*, *Scopolia*, *Schizanthus*)*.* Cocaine and related alkaloid, which are analgesics and CNS stimulants, are produced from leaves of coca (*Erythroxylum coca*). TA are antagonists at the muscarinic acetylcholine receptor and therefore show parasympatholytic properties. These alkaloids block smooth muscles, which leads to spasmolysis and loss of motility in several organs (GI tract, bladder, bronchia), inhibition of glandular secretions (salivary, bronchial, sweat glands), tachycardia, at the eye mydriasis and accommodation disturbance. Hyoscyamine and much stronger scopolamine produce central excitation (with hallucinations), at higher doses a central paralysis is more dominant [11,12,13,23,24,38,52,103].

Plants, extracts and pure tropane alkaloids have a long history of magic and murder. They have been taken since antiquity to generate hallucinations and intoxication [1,31,52,100]. Dried leaves of *Datura* were used formerly as herbal cigarettes to treat patients with asthma and other respiratory conditions. Atropine is been used medicinally for the treatment of spasms of smooth muscles in the gastrointestinal and urinary tract, gall ducts and bronchia. However, also to treat bradycard arrhythmia and hyperhidrosis. Atropine and scopolamine are locally employed at the eye as mydriatic and cycloplegic to facilitate inspections and diagnosis. Hyoscyamine and especially scopolamine are used as premedication for narcosis because of their sedating properties. In case of poisoning with parasympathomimetics atropine is applied as an antidote. Scopolamine is used as transdermal plasters to treat travel sickness [3,11,12,13,23,24,38,52,55].

#### 3.2.2. Non-Protein Amino Acids (NPAAs)

NPAAs occur in seeds, leaves and roots of legumes (Fabaceae) and in some monocots (Alliaceae, Iridaceae, Liliaceae), but also in Cucurbitaceae, Cycadaceae, Euphorbiaceaee, Resedaceae, and Sapindaceae. NPAAs often accumulate in seeds where they serve as herbivore repellent nitrogen storage molecules, which are recycled during growth of the seedling after germination [11,12,13,23,24,38].

The structure of NPAAs, of which more than 700 have been identified, resemble those of the 20 protein amino acids, therefore they can be considered as structural analogues. For example, 3-cyanoalanine is an analogue to l-alanine, canaline to l-ornithine, S-aminoethylcysteine to l-lysine, l-azetidine-2-carboxylic acid to l-proline, albizziine to l-glutamine, Se-methylselenocysteine to l-methionine, and l-canavanine or l-indospicine to l-arginine. NPAAs can inhibit the uptake and transport of amino acids or disturb their biosynthetic feedback regulations. Since ribosomal transfer ribonucleic acid (tRNA) transferases cannot usually discriminate between a protein amino acid and its analogue some NPAAs are even incorporated into proteins, resulting in defective or malfunctioning proteins. Other NPAAs interfere with neuronal signal transduction or enzymatic processes. DNA-, RNA-related processes are inhibited by canavanine and mimosine, collagen biosynthesis by mimosine, or β-oxidation of lipids by l-hypoglycine [11,12,13,23,24,38].

A special case of NPAAs can be found in garlic and onions (*Allium* species); alliin is converted into a reactive metabolite allicin and others that can bind to SH-groups of various proteins (Figure 1). This would explain the wide range of pharmacological activities (antidiabetic, antihypertensive, antithrombotic and antibiotic properties) that were attributed to garlic. Propanethial S-oxide derived from S-propenylcysteine S-oxide occurs in onion (*Allium cepa*) and responsible for the main lachrymatory activity when onions are cut or bruised [11,12,13,23,24,38].

#### 3.2.3. Cyanogenic Glucosides (CG) and HCN

CG are especially abundant in of seeds, leaves and roots of Caprifoliaceae, Euphorbiaceae, Fabaceae, Juncaginaceae, Linaceae, Passifloraceae, Poaceae, Rosaceae, Ranunculaceae, and Sapindaceae. CG are stored in the vacuole as prefabricated defense chemicals (“prodrug” principle). If tissue decomposition occurs due to wounding through an herbivore or a pathogen, then a β-glucosidase comes into contact with the cyanogenic glucosides, which are consecutively split into a sugar and a nitrile moiety that is further hydrolyzed to hydrocyanic acid (HCN) and an aldehyde. HCN is the strong poison as it binds to cytochrome oxidase in the mitochondrial respiratory chain. HCN therefore effectively inhibits mitochondrial respiration and in consequence adenosine triphosphate (ATP) production [11,12,13,23,24,38]. Death is caused by respiratory arrest. Laetrile (termed vitamin B_17_) is an ineffective anticancer drug based on amygdalin, which was widely used in USA, has led to several cases of severe HCN poisoning. TCM uses amygdalin as an antitussive agent [11,12,13,23,24,38].

#### 3.2.4. Glucosinolates and Mustard Oils

Glucosinolates occur in seeds, leaves and roots in the Brassicales (families Brassicaceae, Capparaceae, Moringaceae, Resedaceae, and Tropaeolaceae). The glucosinolates are stored as prefabricated inactive vacuolar defence compounds. When they come into contact with myrosinase, the active mustard oils are released. Mustard oils are highly lipophilic and can disturb the fluidity and permeability of biomembranes and bind to various enzymes, receptors or other macromolecules, such as DNA (thereby exhibiting a substantial antimicrobial effect) (Figure 1). Isothiocyanates are responsible for the distinctive, pungent flavor and odor of mustards and horseradish and are strong irritants to skin, mucosal tissues of mouth, throat and GI tract and eye [11,12,13,23,24,38].

Mustard oils, like the pungent capsaicin activate TRP channels (transient receptor potential family of calcium ion channels). In addition, isothiocyanates are reactive compounds that can form covalent bonds with SH-, NH_2_- groups of proteins (Figure 1). If many proteins are treated in such a way, cells die and inflammation starts, usually resulting in blister formation. Goitrin (5-vinyl-2-oxazolidinethione), which derives from progoitrin in most brassicas, inhibits the incorporation of iodine into thyroxine precursors and interferes with its secretion and can therefore induce the development of goitre. Plants with glucosinolates are often used as spices or vegetables; mustard oils have been employed in traditional medicine to treat rheumatism (topical application) and bacterial infections [11,12,13,23,24,38].

#### 3.2.5. Lectins and Peptides

Lectins are small glycosylated and protease-resistant proteins, which are common in seeds of several plants, such asabrin in *Abrus precatorius*, phasin in *Phaseolus vulgaris*, ricin in *Ricinus communis*, and robin in *Robinia pseudoacacia*. Less toxic lectins occur in seeds of several plants, especially of legumes and mistletoe (*Viscum album*), which has been used in phytomedicine. Some of them contribute to allergic properties of a plant, such as peanut lectin (PNA) in peanut seeds (*Arachis hypogaeia*), ragweed pollen allergen (Ra5) from *Ambrosia elatior*. In plants, seed lectins serve as defence compounds against herbivores and nitrogen storage compounds that are remobilised during germination [11,12,13,23,24,38].

Lectins bind to cells via the haptomer (haemagglutinating activity) and become internalized by endocytosis. Once in the cell they have an affinity for ribosomes and the A-chain (the effectomer, which has *N*-glycosidase activity) blocks ribosomal protein translation by inactivating elongation factors EF1 and EF2. A cell that no longer is able to make proteins will die. Lectins are toxic when taken orally, but more toxic when applied intramuscularly or intravenously. Lectins are among the most toxic peptides produced in nature. Other toxic peptides are found in the venom of snakes, spiders, other animals and in some bacteria (causing whooping cough, cholera, or botulism). Lectins and small peptides can be inactivated by heat; therefore, extensive cooking in water at more than 65 °C usually destroys these toxins. Seeds of several plants accumulate other small peptides such as protease inhibitors. They inhibit the activity of intestinal proteases, such as trypsin and chymotrypsin [11,12,13,23,24,38].

Some plants are rich in hydrolytic proteases, such as bromelain in *Ananas comosus*, ficin in *Ficus glabrata*, papain in *Carica papaya*. They are used medicinally to treat inflammation and digestive problems [11,12,13,23,24,38].

Several small antimicrobial peptides (AMP) are present in many plants but often overlooked in phytochemical analyses. AMP exhibit powerful antimicrobial activities because they can disturb membrane activity in microbes, including multidrug resistant bacterial strains, such as MRSA [68,120]. In combination with antibiotics or antimicrobial SM they can overcome most pathogens [120].

## 4. Conclusions

From a perspective of evolutionary pharmacology, secondary metabolites represent a fascinating library of preselected bioactive compounds with a broad activity towards human cells, bacteria, fungi, viruses and parasites. Some SM appear to be specific for one or a limited number of molecular targets (such as alkaloids, cardiac glycosides) (Table 1) whereas most SM which are present in extracts used in herbal medicine (various phenolics, terpenoids) (Table 2) are multitarget agents modulating the activity of proteins, nucleic acids and biomembranes in a less specific way (Figure 1, Figure 2 and Figure 3). Some SM affect the neuro system of animals, and several of them have been and are still used as stimulants, mind-altering and hallucinogenic drugs.

Nevertheless, also the bioactivities of multitarget drugs can be described in terms of pharmacology and biochemistry. They thus represent rational medicines which can be used to treat a wide range of health disorders, diseases and infections. Apparently, some SM of an extract can interact in a synergistic fashion which would potentiate their bioactivities. This is a fascinating topic which should attract more attention from pharmacologists.

Phytotherapy was and is still used in many countries around the world. For many health conditions and infections, it provides a cost-effective and low-risk alternative to synthetic drugs which often exhibit a wide range of severe side effects.

Using the new tools of molecular cell biology, genetics, immunology and NGS, many bioactivities of SM can be studied in more detail and precision. It is important that phytochemists not only isolate SM and describe their chemical structures but that they also study their biological activities alone or in combinations. In order to translate the findings from various laboratories, we need clinical trials to corroborate the efficacy of herbal drugs and to market them as evidence based medicines.
